# Editorial: Cancer diagnosis and novel drug discovery based on microbiome

**DOI:** 10.3389/fmicb.2023.1144986

**Published:** 2023-05-10

**Authors:** Peng Chen, Ge Zhang, Jianhui Wang

**Affiliations:** ^1^School of Pharmacy, Lanzhou University, Lanzhou, Gansu, China; ^2^Department of Microbial and Biochemical Pharmacy, School of Pharmaceutical Sciences, Sun Yat-sen University, Guangzhou, China; ^3^Department of Pathology, Yale University, New Haven, CT, United States

**Keywords:** microbiome, high throughput sequencing, cancer, diagnosis, drug discovery

The human microbiome comprises more than 40 trillion microbes and it consists of over 3,000 species, which are mainly distributed in the intestinal tract, oral cavity, and skin. The microbiota contains abundant genetic and metabolic diversity, thus being closely related to our health and considered as the second human genome. Especially, in the last decades, advances in high throughput sequencing gradually reveal the vital roles of microbiome in the initiation, development, metastasis, prognosis, patient stratification, and clinical therapeutics of various cancers. Besides, *Helicobacter pylori* and many other bacteria, such as *Fusobacterium nucleatum*, pks+ *Escherichia coli*, and Enterotoxigenic *Bacteroides fragilis*, were verified to participate in the deterioration of cancer and affect the chemotherapy efficacy and toxicity by experiments *in vitro* and *in vivo*. Consequently, targeting these oncogenic bacteria to explore novel antibacterial and anticancer agents will benefit patients. With the approval of fecal transplantation therapy for *Clostridium difficile* infection, novel therapies including probiotics, prebiotics, phage, engineering bacteria, and dietary regulation also show great application potential, complementing the drug bank of chemical-lead compounds and natural products.

Though numerous omics data were produced, the mechanism of the microbiota and related drug discovery remains undefined. The main aim of this Research Topic was to unveil the roles of the human microbiome in various cancers using clinical samples, bioinformatics analysis, and epidemiological statistics, and to uncover the potential of disease treatment by modulating microbiota. Four articles have been published on this Research Topic, expanding our knowledge on the cancer progression, metastasis, micro-environment, and curative effect from the aspect of the microbiome.

Yu et al. collected fecal samples of healthy people (NCs), breast cancer patients with no metastasis (BNs), and breast cancer patients with bone metastasis (BMs), then explored variations in the gut microbiota using 16S rRNA sequencing. They found that alpha diversities, such as observed species, Chao and ACE indexes, gradually decreased in NCs then BNs and BMs. *Streptococcus, Campylobacter*, and Moraxellaceae were more abundant in BNs and BMs, while *Megamonas* and *Akkermansia* were depleted in the BMs, which might be related to bone metastasis. Additionally, it was predicted that lipid transportation and metabolism and folate biosynthesis participated in breast cancer occurrence and that steroid hormone biosynthesis influenced bone metastasis. As the first report of gut microbiota and breast cancer bone metastasis, the mechanism of gut microbiota regulating bone homeostasis needs further research.

Due to the difficulty of collecting clinical biopsies according to strict inclusion and exclusion criteria, studying the microbial composition of tissue samples and their associations with diseases remains challenging. Analyzing published multi-omics data from public databases with statistics and bioinformatics is an alternative strategy. Yang et al. acquired information on esophageal squamous carcinoma (ESCC) from The Cancer Genome Atlas (TCGA) and The Cancer Microbiome Atlas (TCMA) databases and classified the tissue-resident micro-environment of ESCC into two clusters, with Cluster A (higher proportion of certain tissue-resident microbiota) and Cluster B (lower proportion of certain tissue-resident microbiota) corresponding to comparatively better and worse survival, respectively. Additionally, differentially expressed genes, functional annotation, and somatic genomic mutations demonstrated the impact of discrepant esophageal tissue-resident microbiota on the tumor.

To better understand the roles of microbiota and other risk factors in the progression of cancer and to implement interventions, Chen et al. used the adjusted logistic regression model to assess the risk factors between each of two consecutive stages based on the Wuwei Cohort [including 1,739 enrolled patients with chronic non-atrophic gastritis (no-CAG); 3,409 patients with chronic atrophic gastritis (CAG); 1,757 patients with intestinal metaplasia (IM); 2,239 patients with low-grade dysplasia (LGD); and 208 patients with high-grade dysplasia (HGD) or gastric adenocarcinoma (GAC)]. They concluded that *H. pylori* infection triggered a multistep process. From no-CAG, CAG, to IM, *H. pylori* provided the seed of the cascade leading to GAC. Therefore, apart from important influences of genetic and environmental factors, patients may benefit from the control and eradication of *H. pylori*, thus decreasing the incidence of GAC.

Taking the crucial effects of microbiota into consideration, restoring the dysbiosis of local microbial ecology to alleviate disease is promising. Zhao et al. investigated the antiaging effects of parishin, a phenolic glucoside isolated from traditional Chinese medicine *Gastrodia elata*. They found that parishin ameliorated aging-induced cardiopulmonary fibrosis and decreased the p16^*Ink*4*a*^, GDF15, and IL-6 levels. Furthermore, after parishin treatment, the abundance of opportunistic pathogenic bacteria such as *Turicibacter* and *Erysipelatoclostridium* decreased, while the *Prevotellaceae* NK3B31 group and *Rombousia* were enriched. Accordingly, the altered gut microbial function was mainly associated with sugar, lipid, amino acid, and nucleic acid metabolism, which was partially corroborated by fecal metabolome analysis. This study provides a theoretical basis for the development of parishin against aging.

In conclusion, it is vital to elucidate the changes and mechanisms of the human microbiome in the advances of cancers before carrying out clinical transformation and application. In the future, more efforts should be focused on the following directions. First, to obtain robust characteristics of microbial changes, we need not only a larger cohort comprising patients with different backgrounds but also an independent validation cohort. Second, higher resolution sequencing technology such as metagenomic sequencing plus multi-omics data need to be integrated to figure out specific species or sub-species. Third, analyzing the human microbiome is an integrated process from sampling to bioinformatics analysis, sometimes making it hard to obtain similar results to the original sequencing studies. Thus, unifying the protocols and *in silico* decontamination methods is of utmost significance. The International Human Microbiome Standards (IHMS) project has taken an important step in the development of standard operating procedures. Fourth, with the rapid advancement of next-generation sequencing since 2005, tremendous data have been generated, including high-quality and comprehensive sequencing projects (e.g., Human Microbiome Project, HMP). A meta-analysis by reusing these available resources will improve synergy, minimize unnecessary overlap, and finally foster overall progress in human health and pharmaceutical research. Fifth, defining the causal relationship between microbes and cancers remains challenging, yet new advances in gene editing and mathematical modeling provide us with powerful tools. Sixth, most current studies are focused on bacteria, whereas fungi and viruses are of interest, and their roles in human cancers as well as interactions with bacteria or host cells have remained unexamined. Finally, targeting these microbes, precise modulation with beneficial bacteria and novel drugs is still in the infancy stage.

## Author contributions

PC, GZ, and JW were joint co-editors of this Research Topic. All authors contributed equally to the article and approved the submitted version.

